# Forest response and recovery following disturbance in upland forests of the Atlantic Coastal Plain

**DOI:** 10.3389/fpls.2014.00294

**Published:** 2014-06-26

**Authors:** Karina V. R. Schäfer, Heidi J. Renninger, Nicholas J. Carlo, Dirk W. Vanderklein

**Affiliations:** ^1^Department of Biological Sciences, Rutgers University NewarkNewark, NJ, USA; ^2^Earth and Environmental Science Department, Rutgers University NewarkNewark, NJ, USA; ^3^Department of Biology and Molecular Biology, Montclair State UniversityMontclair, NJ, USA

**Keywords:** forest disturbance, physiology, forest response, modeling, oaks, pine

## Abstract

Carbon and water cycling of forests contribute significantly to the Earth's overall biogeochemical cycling and may be affected by disturbance and climate change. As a larger body of research becomes available about leaf-level, ecosystem and regional scale effects of disturbances on forest ecosystems, a more mechanistic understanding is developing which can improve modeling efforts. Here, we summarize some of the major effects of physical and biogenic disturbances, such as drought, prescribed fire, and insect defoliation, on leaf and ecosystem-scale physiological responses as well as impacts on carbon and water cycling in an Atlantic Coastal Plain upland oak/pine and upland pine forest. During drought, stomatal conductance and canopy stomatal conductance were reduced, however, defoliation increased conductance on both leaf-level and canopy scale. Furthermore, after prescribed fire, leaf-level stomatal conductance was unchanged for pines but decreased for oaks, while canopy stomatal conductance decreased temporarily, but then rebounded the following growing season, thus exhibiting transient responses. This study suggests that forest response to disturbance varies from the leaf to ecosystem level as well as species level and thus, these differential responses interplay to determine the fate of forest structure and functioning post disturbance.

## Introduction

In recent decades the importance of disturbances on the forest carbon and water cycles have been recognized as well as the effects of climate change in modulating these (Dale et al., 2001; Kurz et al., 2008a; Reichstein et al., 2013; Gatti et al., 2014). Future predictions of forest recovery and health depend on an understanding of current mechanisms of mortality and an understanding of forest structure, function, and underlying mechanisms of species compositional dynamics under disturbance regimes (Seidl et al., 2011a). To date, most models do not take into account physiological changes, trade-offs in response to multiple **forest disturbances** (physical and biogenic), feedback mechanisms between nutrients and forest species, or potential species shifts (Dietze et al., 2011, 2013; Medvigy et al., 2012; Richardson et al., 2012). In addition, mechanisms of mortality are not well understood and thus not incorporated into models (McDowell et al., 2008, 2011). Ecosystem response to extreme climate events such as drought can result in increases in defoliation, fire or wind-throw (Ayres and Lombardero, 2000; Dale et al., 2001; Reichstein et al., 2013) and a decrease in transpirable soil water content (Klein et al., 2014). Forest functioning and species composition will likely be altered by re-occurring droughts, insect infestations and windthrow, while the changes in energy partitioning will likely have impacts for regional climate in forest ecosystems (Roy and Avissar, 2002). This, in turn, could increase fire risk (Smithwick et al., 2009; Seidl et al., 2011b; Stephens et al., 2013). Conversely, climate extremes can have delayed feedback impacts on soil water content, and thus, ecosystem function (Reichstein et al., 2013). Additionally, species may vary in their responses to such occurrences (Schäfer, 2011). Therefore, specific ecosystem responses are not well known and are difficult to model due to a lag in response (Reichstein et al., 2013).

KEY CONCEPT 1 **Forest disturbance**

Any physical or biogenic agent that disrupts the structure and function of forests, such as windthrow, insect pests or pathogens on an ecosystem scale.

Clearly, in order to build predictive models, the processes need to be captured on the leaf and/or canopy scale. While canopy net assimilation scaled via sapflux (see Schäfer et al., 2010) *vis a vis* gross ecosystem production measured with eddy covariance (see analysis in Amiro et al., 2010) show overall reduced carbon uptake after insect attack, the process on the leaf-level shows compensatory responses such as higher photosynthetic activity (Heichel and Turner, 1983; Vanderklein and Reich, 1999) or water use per unit leaf area (Meinzer and Grantz, 1991; Schäfer, 2011); even under drought conditions (Hawkes and Jon, 2001). However, nutrient removal *via* defoliators could reduce photosynthetic capacity over time, thus effectively hindering recovery (Krause and Raffa, 1996). Therefore, the overall reduction at the canopy scale is mediated through leaf-level compensations rather than just a function of reduced leaf area as it is implemented in models (see Medvigy et al., 2012). In contrast, **prescribed fires** have only short-term effects on overstory trees or the ecosystem at large (Clark et al., 2012; Renninger et al., 2013), given that they mainly affect understory shrubs and forest floor fuel loading (Boerner, 1981; Boerner et al., 1988). Any effect on overstory trees or ecosystem scale carbon and water cycling are transient (Clark et al., 2012; Renninger et al., 2013). Wildfires, however, have a devastating effect on the water and carbon balance of forests, as they often are stand replacing or largely more destructive to overstory trees (Hurteau and North, 2008, 2009; Hurteau et al., 2008, 2011; Wiedinmyer and Hurteau, 2010). Furthermore, these wildfires as disturbance regimes can potentially play a huge role in forest health and structure (Heinselman, 1973). Depending on the burn regime, fires can lead to both horizontal and vertical structural changes by altering canopy gaps, species composition, and tree densities, which can then subsequently alter competitive relationships (Heinselman, 1978; Boerner et al., 1988). It has also been found that fire can have a direct impact on physical and chemical properties of the soil (Granged et al., 2011), which could potentially lead to altered physiological responses of the overstory trees, further affecting the carbon and water budgets. Thus, insight into hydrodynamics (Lopushinsky and Klock, 1980; Bohrer et al., 2005; Thomsen et al., 2013), nutrient limitation (Lovett and Tobiessen, 1993; Krause and Raffa, 1996; Vanderklein and Reich, 2000) or enhancement of photosynthetic capacity (Heichel and Turner, 1983; Haukioja et al., 1985; Hodgkinson, 1992; Vanderklein and Reich, 1999) in response to disturbances such as drought, insect defoliation, and fire would help build better predictive models to assess forest structure, function, and species compositional shifts under disturbance regimes. This will help improve predictions of water and carbon cycling of forest ecosystems.

KEY CONCEPT 2 **Prescribed fire**

Management practice to reduce fuel load (forest floor litter and understory brush) in order to prevent wildfires.

Capitalizing on a long-term data collection effort in a xeric forest of the Atlantic Coastal Plain, the New Jersey Pine Barrens, insights into drought and prescribed fire (as a physical forcing agent) and insect defoliation (as a biogenic forcing agent) plant responses have improved our understanding of plant compensatory responses, potential mortality agents and species compositional shifts, thus enhancing predictions of water and carbon cycling of forests (Schäfer et al., 2010, 2013; Schäfer, 2011; Clark et al., 2012; Medvigy et al., 2012, 2013; Renninger et al., 2014b). It is important to note differences in physical disturbances, such as fire and windthrow that are non-species specific and biogenic disturbances, such as defoliators or phloem feeders that are species specific and thus have a larger impact on forest dynamics and species compositional changes. Here, we provide a synthesis and insights of the effects of physical and biogenic disturbance to water and carbon cycling in upland forests of the New Jersey Pine Barrens.

## Materials and methods

### Site description

**Figure 1:**
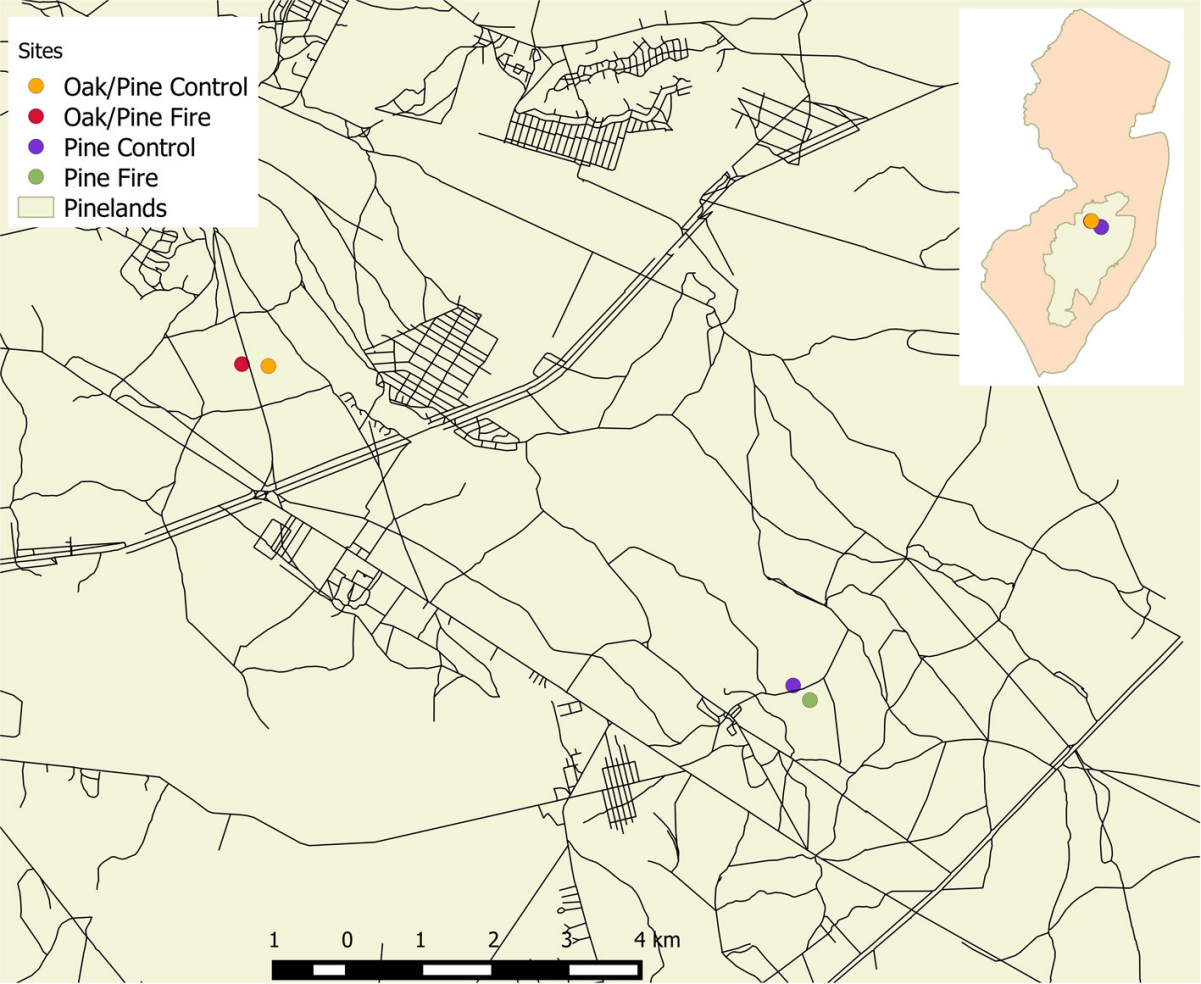
**Map of New Jersey (insert) with the New Jersey Pine Barrens highlighted in the center**. Large map shows the oak/pine sites and the pine site. The long-term site is designated in orange (see also description in text).

For this study, a long-term research site in an upland oak/pine forest in the New Jersey Pine Barrens was chosen that had a nearby prescribed fire site about 800 m away, and two pine stands, one prescribed fire and one control site, that we reported about earlier (Renninger et al., 2013), which is about 8 km due south-east from the long-term study site (see Figure 1). The sites are located in the New Jersey Pine Barrens in southern New Jersey (see Figure 1) with primarily sandy soil with characteristic low nutrient retention and water holding capacity (Schäfer, 2011). In the upland oak/pine forest in the Brendan T. Byrne State Forest (see Figure 1, N 39° 55′ 0″, W 74° 36′ 0″), the dominant tree species are *Quercus prinus* Willd. (chestnut oak), *Q. velutina* Lam. (black oak), and *Q. coccinea* Münchh. (scarlet oak), with scattered *Q. stellata* Wangenh. (post oak), and *Q. alba* L. (white oak), *Pinus rigida* Mill. (pitch pine), and *P. echinata* Mill. (shortleaf pine). The upland pine and pine/oak forest primarily consists of *P*. *rigida* with scrub oak (*Q. ilicifolia* Wagenh., *Q. marlandica* Muenchh.) in the understory (Clark et al., 2012; Renninger et al., 2013). At the long-term experimental stand at the oak/pine upland forest, a drought was observed in August of 2006 and in July of 2010, as well as a total gypsy moth defoliation in June 2007 of 21% of the upland forest in the NJ Pine Barrens and a partial defoliation in 2008 (Schäfer et al., 2010). After the defoliation event in 2007, the canopy re-flushed with 50% of the leaf area observed in previous years at the peak of the season. The prescribed fire at the pine and oak/pine sites occurred in March of 2011 and March 2012, respectively.

### Environmental data

In order to calculate vapor pressure deficit (VPD) of forest air, environmental measurements such as air temperature (*T*_air_) and relative humidity (*RH*, HMP45C Vaisala, Helsinki, Finland) were made about two-thirds of the canopy at the respective experimental sites. Air temperature and relative humidity were used to calculate vapor pressure deficit of the air (VPD) according to Goff and Gratch (1946). In addition, precipitation throughfall (*P*_T_, TE525, Texas Electronics Inc, TX, USA), and soil moisture from 0 to 30 cm (Θ m^3^ m^−3^, CS616, Campbell Scientific, Inc, Logan, UT, USA) were recorded every half-hour using data loggers (CR3000 or CR1000, Campbell Scientific Inc, Logan, UT, USA). These measurements are continuous at the long-term oak/pine site and were conducted at the control pine site throughout the study period (Renninger et al., 2013).

### Leaf-level measurements

In order to measure leaf-level physiological responses, leaf-level net photosynthesis and leaf stomatal conductance were measured with a Licor 6400 XT with a red/blue light source attached (LiCor Bioscience Inc., Lincoln, NE, USA) before and after drought in the upland oak/pine forest (Schäfer, 2011), before and after prescribed fire at a burned and a control plot in the upland pine forest (Renninger et al., 2013) and in 2012 and 2013 at the oak/pine stand at the long-term study site and at the prescribed fire site close by (see Figure 1). The prescribed fire at the oak/pine site was conducted in March 2012, thus results presented here, are the first and second growing season after the fire. The conductance measurements were performed at 400 ppm external CO_2_ concentration and at light saturating conditions (>1500 μmol m^−2^ s^−1^).

### Canopy stomatal conductance

Canopy-level transpiration can be measured *via* sapflux and scaled to canopy stomatal conductance (Schäfer et al., 2010). This was done in five to seven *Quercus prinus*, and five to seven *Q. velutina* in the long-term study stand and four individuals each in the second stand, which underwent a prescribed fire in March 2012 (Renninger et al., 2014b), and in two *Q. alba* and in three *Pinus rigida* at the oak/pine upland forest. At the pine site, eight individuals of *P. rigida* were chosen for sapflux measurements at each of the prescribed fire and control sites (Renninger et al., 2013). Details about the setup and scaling for the upland oak/pine sites can be found in Renninger and Schäfer (2012) and for the pine site in Renninger et al. (2013). Briefly, sapflux is scaled to canopy transpiration by multiplying with sapwood area per unit ground area and to canopy transpiration per unit leaf area by multiplying with sapwood area per unit leaf area per individual (pine) and of the canopy per species (oaks). Sapwood area was measured from tree cores and a relationship with canopy leaf area derived with diameter at breast height (Renninger et al., 2013, 2014b). In order to scale to canopy stomatal conductance, transpiration per unit leaf area is divided by VPD assuming the canopy is well coupled to the atmosphere and the water in storage contributing to transpiration accounted for by lagging the driving force (VPD) to transpiration (Schäfer et al., 2010; Schäfer, 2011).

### Biometric measurement

Every year, at the end of the growing season, diameter at breast height (dbh) was measured in the experimental plot in the upland oak/pine forest comprising 0.3 ha and the nearby fire plot comprising 0.0225 ha. For the prescribed fire experiment in the upland pine forest, two experimental plots were established, each 0.0225 ha in size and dbh measured for all trees in the plot. Using allometric relationships derived by Whittaker and Woodwell (1968), leaf area was determined for scaling purposes (see above) or measurements of light transmission (LAI 2000) were conducted for the oaks to determine leaf area (Renninger et al., 2014b).

### Statistical analysis

Comparisons of leaf- and canopy-level stomatal conductance between fire and control sites were made using ANOVA in R version 2.5.1 (The R Foundation for Statistical Computing; http://www.R-project.org). *P*-values less than 0.05 were deemed significant.

## Results

Measurements of transpiration, ecophysiological parameters, biometric variables and eddy covariance measurements in an oak/pine ecosystem in the Atlantic Coastal Plain (New Jersey Pinelands) showed a relative conservatism of water use (Clark et al., 2012) on an ecosystem level, but longer lasting effects on carbon balance after insect defoliation. While post-defoliation (2012) transpiration and evapotranspiration are similar to pre-defoliation levels (2006), post-defoliation carbon fluxes have not returned to pre-disturbance levels after 5 years of recovery due to a 25% reduction in basal area following tree mortality (Schäfer et al., 2013). Defoliation frequency also affects recovery, with modeled carbon fluxes under various defoliation scenarios showing pronounced reduction in productivity under frequent defoliation, but no effect if defoliation occurs at a rate of >15 years (Medvigy et al., 2012).

Despite a relatively consistent seasonal water use through various disturbances, defoliation and drought affected water use differently. For example, canopy transpiration (E_C_) after defoliation and subsequent re-sprouting, was reduced by 25% compared to pre-defoliation levels, even though only half of the leaf area was replaced. However under severe drought conditions in 2006 and 2010, E_C_ was only reduced by 8 and 18% respectively (Table 1, Schäfer et al., 2013). Therefore, prolonged drought had a lesser effect on E_C_ than reduced foliage or episodic defoliation, suggesting these trees have access to deeper soil moisture. These data also suggest that defoliation may make trees more sensitive to drought over time as evidenced by the higher reduction of E_C_ during a 2010 drought period (post-defoliation) compared to a 2006 drought (pre-defoliation) (Schäfer et al., 2013).

**Table 1:** **Summary of responses to disturbances in the New Jersey Pine Barrens**.

	**A_net_**	**A_nC_ GEP**	**g_S_**	**G_C_**	**LAI**	**Leaf N**	**Soil N**	**Soil CO_2_**
Defoliation	↑	↓	↑	↑	↓	↓	?	±
Drought	↓	↓	↓	↓	±	±	±	?
Prescribed fire	±	±	±	±	±	±	±	±

A_net_, net assimilation at the leaf level; A_nC_, canopy net assimilation; GEP, gross ecosystem production; g_S_, stomatal conductance at the leaf level; G_C_, canopy stomatal conductance; LAI, leaf area index; Leaf N, leaf nitrogen concentration; soil N, soil nitrogen concentration; soil CO_2_, soil carbon dioxide efflux; ↑, increase; ↓, decrease; ±, no change; ?, not known. Details and references see text.

Differential physiological responses of the various oak species as well as pitch pine may also create a species shift in an ecosystem that is also prone to fire (Table 2). In this ecosystem, *Quercus prinus* showed consistently lower stomatal conductance, photosynthesis and maximum carboxylation rate compared to *Quercus velutina*, however both oak species displayed similar **water and nutrient use efficiencies** (Renninger et al., 2014b). Likewise, *Pinus rigida*, a predominant species in the Pinelands, showed comparable water– and nutrient use efficiencies to the oak species investigated signifying similar strategies in this ecosystem with respect to their efficiencies. However, *Q. velutina* had higher mortality rates than *Q. prinus* suggesting a possible shift in oak species with more frequent defoliation events (Schäfer, 2011). Likewise, *P. rigida* may be released from competition if more oaks species face mortality due to gypsy moth defoliation occurrences (Medvigy et al., 2012).

**Table 2:** **Comparison of leaf- and canopy-level stomatal conductance in an oak-pine forest that experienced a prescribed fire**.

	**Leaf-level stomatal conductance (mol m^−2^ s^−1^)**			**Canopy-level stomatal conductance (mol m^−2^ s^−1^)**		
	**Fire**	**Control**	***P*-value**	**Fire**	**Control**	***P*-value**
**YEAR OF FIRE GROWING SEASON**						
*Pinus*	0.072 (0.0099)	0.11 (0.018)	0.11	0.10 (0.0023)	0.12 (0.0019)	**<0.001**
*Q. alba*	0.17 (0.011)	0.28 (0.033)	**<0.001**	N/A		
*Q. prinus*	0.14 (0.031)	0.23 (0.013)	**0.05**	0.071 (0.0035)	0.11 (0.0031)	**<0.001**
*Q. velutina*	0.23 (0.019)	0.41 (0.043)	**<0.001**	0.089 (0.0034)	0.13 (0.005)	**<0.001**
**ONE YEAR POST-FIRE**						
*Pinus*	0.16 (0.019)	0.25 (0.01)	0.18	0.18 (0.028)	0.19 (0.011)	0.74
*Q. alba*	0.32 (0.017)	0.22 (0.017)	0.14	N/A		
*Q. prinus*	0.21 (0.020)	0.20 (0.017)	0.75	0.10 (0.0055)	0.13 (0.0052)	**0.008**
*Q. velutina*	0.36 (0.044)	0.42 (0.023)	0.2	0.12 (0.011)	0.18 (0.018)	0.08

P < 0.05 are shown in bold.

KEY CONCEPT 3 **Water use efficiency**

Amount of carbon dioxide uptake per unit water lost, or per stomatal conductance.

KEY CONCEPT 4 **Nitrogen/Nutrient use efficiency**

Amount of carbon dioxide uptake per unit nitrogen in the leaf or per unit nitrogen per unit area of the leaf.

**Figure 2:**
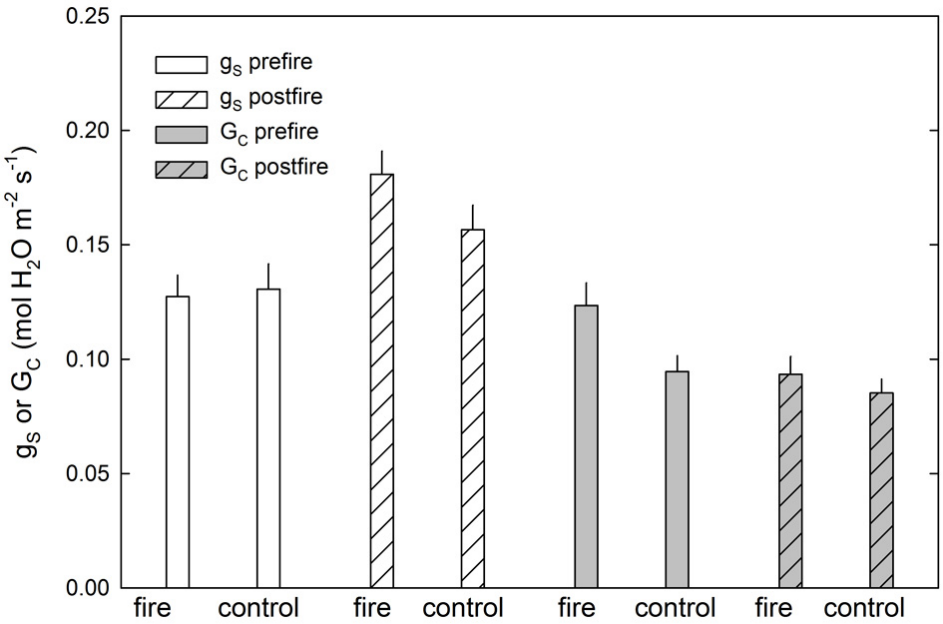
***P. rigida* canopy and leaf level stomatal responses to prescribed fire at the 2011 fire site, Brendan T Byrne Forest, see also Renninger et al. (2013)**.

Prescribed fire in this ecosystem had a short-term effect on leaf-level and canopy-level stomatal responses (Figure 2, Table 2). Leaf-level stomatal responses remained unchanged in relation to the coupled control site for *P. rigida*, directly following the fire (Figure 2). Comparing the pine site with the upland oak/pine site, increased water use by overstory pines was observed, while at the oak/pine site, the fire decreased stomatal conductance the summer after the fire. Therefore, there could be differing effects depending on stand type with the pine-dominated stand being positively affected by fire and the oak-dominated site being negatively affected. For example, pre-fire canopy stomatal conductance (G_c_) at the pine fire site was significantly higher than the control site (*P* = 0.01). However, following the fire, the control and fire site exhibited no statistical difference (*P* = 0.3). In this forest ecosystem, prescribed fire, therefore, has little effect on the leaf-level physiological responses of overstory pitch pines (Figure 2, Table 2). While some initial trends were noticed in discrete cases, these responses did not hold true across the two prescribed fires sites and thus show differential responses across different stands with different species composition. However, a common trend that did seem to develop was a transient response to a prescribed fire. In some cases, such as carboxylation efficiency and maximum assimilation with respect to increase in carbon dioxide concentration, there was an initial increase following the fire, which subsided by the first or second summer after the fire (Renninger et al., 2013). Another common trend was a delayed response in which physiological differences from late winter/early spring prescribed fires were not seen until the summer growing season. For example, no initial increase in either photosynthetic capacity (V_cmax_) or intrinsic water use efficiency was noted in the weeks post fire, indicating a lag of response until new needles are produced capitalizing on release of nitrogen post-fire. However, a large increase in these two parameters was measured by the summer growing season. These trends suggest that prescribed fires affect stands differently depending possibly on fire intensity, fuel loading and species composition.

## Discussion

The major knowledge gap to understand and thus model disturbance, recovery and resilience are that most terrestrial or demographic vegetation models [such as BIOME-BGC (Running and Gower, 1991), ED2 (Medvigy et al., 2009) etc.] do not take into account disturbances such as fire, insect defoliation, hurricane or snow load disturbance (McCarthy et al., 2006) and their physiological impacts. In addition, physiological responses in general and parameterization thereof are ill defined in models (Rogers, 2014). Defoliation, for example, has only been implemented through leaf area reduction, but does not take into account compensatory photosynthetic responses (Medvigy et al., 2012). Since, often, photosynthetic capacity (Rogers, 2014) or stomatal conductance are ill-defined in these models [meteorological driven models such as the Ball-Berry Model, (Ball et al., 1987; Medvigy et al., 2013)], it is difficult to incorporate changes due to disturbances that have physiological effects (see Table 1) that are known to be important (Thornton et al., 2002; Rogers, 2014). Likewise, species compositional changes are unknown after disturbance, and at the ecosystem level, responses may be delayed and cannot be measured until years after a disturbance or extreme climatic event (Boerner, 1981; Runkle, 1981, 2000; Reichstein et al., 2013). In addition, recent reports suggest that the Southern Pine Bark Beetle will invade the NJ Pine Barrens potentially increasing mortality to pine species (Gillis, 2013). Therefore, future species composition in this forest depends on a range of insect disturbances, which are driven by climate change making the species dominance outcome unclear. Generally, species composition after physical disturbance changes very little, as the forest gaps are filled with species already present (Runkle, 1981, 1982, 1984; Frelich and Reich, 1999). However, as biogenic disturbances are more species specific, the dynamics are less clear (Kurz et al., 2008b; Seidl et al., 2011a).

Measured plant compensatory responses can confound ecosystem level responses to disturbances, particularly if they lead to delayed responses (Sala et al., 2010). In addition, release from competition can confound or enhance plant physiological responses to disturbances (Wickman, 1980; Runkle, 1981; Runkle and Yetter, 1987; Tilman et al., 1997; Frelich and Reich, 1999; Vanderklein and Reich, 1999). Plant compensatory responses have been well documented and are similar to our findings (Reich et al., 1993; Vanderklein and Reich, 1999; Clinton et al., 2011; Schäfer, 2011), however the incorporation into models is still lacking. Thus, devising strategies to manage forests are yet hampered by this limitation (Seidl et al., 2011a). In addition, the effects of disturbances are not necessarily perceived in a matter of years but rather decades (Baker, 1941) with potentially compounding effects (Stevens and Beckage, 2009; Gaylord et al., 2013; Schäfer et al., 2013). Even if management of disturbances is implemented, such as species compositional changes, the outcome may take decades to take effect (Seidl et al., 2008, 2009).

In the case of prescribed fire (Table 2, Figure 2), the pine site has been positively effected by the prescribed fire, because of a thicker duff layer, surface roots and microbes may have not been damaged (Boerner, 1981; Boerner et al., 1988). However, at the oak/pine site, a thinner duff layer may have led to higher temperature effects at the soil surface, thus potentially damaging some of the surface roots and microbes (Varner et al., 2009). Therefore, plant functional types play an important role in the structure and function of these forests. However, since the responses are short-term and transient (Clinton et al., 2011), the long-term impact on the carbon and water cycling in these forest ecosystems are likely to be small.

While there may be a general framework to assess tree mortality in response to drought and insects (McDowell et al., 2011), ecosystem responses to drought and insect attack (folivory or phloem feeding) are contingent on individual tree trade-offs, which are themselves contingent on tree ontogeny. Barbeta et al. (2003) found that larger trees survived a long-term drought treatment better than smaller trees, presumably because as the smaller trees died, they freed up soil moisture for the larger trees, which may have a combination of deeper root systems and a higher water storage capacity. However, the mortality of the smaller trees must be the result of trade-offs between growth and ability to respond to drought. If smaller trees have higher root to shoot ratios (Kearsley and Whitham, 1989; Boege and Marquis, 2005), yet are more susceptible to drought, then carbon stores and the ability to utilize those stores must be more important for survival than drought resistance *per se*. On the other hand, higher resistance to drought may result in lower maximum assimilation and water use efficiencies (Limousin et al., 2010). Interestingly, this may not be the case in xeric environments, such as the New Jersey Pine Barrens in the Atlantic Coastal Plain investigated here (Schäfer, 2011; Renninger et al., 2014b). Furthermore, larger trees and trees growing in arid regions have larger non-structural carbohydrate pools (Sala et al., 2010) suggesting that they should be less vulnerable to mortality as a result of carbon starvation. Likewise, results from defoliation research using seedlings show that trees may retain a minimum amount of carbon regardless of defoliation intensity (Chapin et al., 1990; Reich et al., 1993; Vanderklein and Reich, 1999). Thus, a distinction needs to be made between total carbon pools and available carbon pools (McDowell et al., 2011).

The interactions and possible trade-offs between tree responses to insect attack and drought are unknown (Agrawal, 2007; Jactel et al., 2012), whereby carbon used for defense against insects cannot be used for repair (i.e., of cavitation induced by drought). Plants may also reduce their carbon demand by reducing respiration rates and/or shedding plant parts in response to drought (Sala et al., 2010). Functionally, shedding plant parts should be similar to defoliation depending on what is shed. On the other hand, a possible trade-off for increased drought resistance could be higher susceptibility to insect attacks (Mattson and Haack, 1987). However, as was also shown here in an upland oak/pine forest in NJPB, *Q. prinus* not only withstood drought better, but also sustained less mortality after gypsy moth disturbance compared to *Q. velutina* (Schäfer, 2011). The differences in mortality may be due to different resource use strategies, whereby *Q*. *velutina* was shown to have higher photosynthetic capacity and nitrogen (N) per unit leaf area, thus was more vulnerable to N removal through insects (Renninger et al., 2014b). Thus, as has been shown before, different species respond differently to drought (Schäfer, 2011; Wu et al., 2011; Renninger et al., 2014b) and may adapt over time to it (Wu et al., 2011) or may become more susceptible to drought over time (Hacke et al., 2001; Anderegg et al., 2013). However, ecosystem function depends not only on biotic or abiotic factors but also sociological and economic factors. The New Jersey Pine Barrens are managed forests in relatively close proximity to large, urban centers, thus how they are managed has consequences for ecosystem processes that can also affect carbon and water dynamics as was shown here with regard to prescribed fires. However, adaptive management practices may take decades to have a perceptible impact (Seidl et al., 2009), thus a forward leaping approach is needed that allows proper management decisions to be made since corrective measures will be difficult (Seidl et al., 2008).

Forest management practices have to be persistent in order to recover forest health (Seidl et al., 2008; Gormley et al., 2012), but also need to be able to address forest mortality, and thus loss in carbon sequestration potential (McCarthy et al., 2006). Research will need to improve our understanding of a) species responses to a particular disturbance, b) mechanisms leading to mortality and c) how to include this mechanistic understanding into models that, in turn, will help to predict future changes and responses of forests. As this study suggests, forest response to disturbance varies from the leaf to ecosystem level as well as species level and thus, these differential responses interplay to determine the fate of forest structure and functioning.

## Conclusions

Forest functioning will likely be altered by re-occurring droughts, gypsy moth defoliation and windthrow of already weakened trees. However, prescribed fire has only transient responses to the carbon and water balance in this ecosystem. In this forest ecosystem, precipitation variations exerted an overriding effect on the hydrological budget compared to biological changes in this forest, thus it is likely that climate change will cause more changes to the groundwater table and therefore water supply to regional populations. However, changes in energy partitioning due to canopy gaps after mortality will likely have impacts for regional climate in forest ecosystems. Also, in a study on snags and coarse woody debris, carbon pools that quadrupled after gypsy moth-drought mortality suggests that, in a back of the envelope calculation, it will take at least 18 years before current dead wood will have respired, making the carbon balance in this forest uncertain (Renninger et al., 2014a). Thus, while the water balance in this forest ecosystem seems to recover faster within this ecosystem (Clark et al., 2012), the carbon balance has still not recovered to pre-defoliation levels. However, prescribed fire has only transient responses to the carbon and water balance in this ecosystem. Gaining a better understanding and developing a mechanistic underpinning of these responses and incorporating them into larger scale models to improve carbon and water cycle modeling is essential (Dietze et al., 2013). Of particular importance is the ability to incorporate into models the physiological responses on the leaf level and potential compensatory responses on the ecosystem level or *vice versa*.

### Conflict of interest statement

The authors declare that the research was conducted in the absence of any commercial or financial relationships that could be construed as a potential conflict of interest.
